# When Immune Defenses Turn Traitor

**DOI:** 10.1371/journal.pbio.0020446

**Published:** 2004-11-30

When pathogens enter your body, most wind up engulfed (by phagocytes), poisoned (by stomach acids), or flushed out of your system. These defenses kick in when a bacterium's toxic secretions bind to cellular receptors that trigger a chain of events ending in an innate response. A wide variety of cells and chemicals, including phagocytes and cytokines, initiate the inflammatory reaction that redirects innate defenses in the bloodstream to the infected site.

If all goes well, the innate system successfully contains and eliminates a pathogen at the site of infection. But if infection persists, so will the inflammatory response, and these first responders turn traitor. It's thought that the immune response rather than the bacteria, for example, causes diarrhea in food poisoning. And when infection leads to death—as happens in septic shock—the immune response may be just as culpable as the infectious microbe.

To better understand the interplay between pathogen and host in the onset of infection and disease, David Schneider and colleagues turned to the genetically compliant fruitfly Drosophila and the food-borne pathogen Salmonella. Working with mutant strains of both fly and bacteria, the authors identified genes important to the development of infection and disease and showed that the host's reaction can indeed be lethal.

Pathogens possess various means to infect their host, unleashing toxins and secreting molecules that enhance virulence by breaching cell membranes and altering the intracellular environment. Fruitflies combat these intrusions with various innate responses, including phagocytosis. In this study, the authors investigated how Drosophila phagocytes find, engulf, and kill invading microbes and then alert the rest of the immune system—and how Salmonella circumvents these defenses to initiate disease.[Fig pbio-0020446-g001]

**Figure pbio-0020446-g001:**
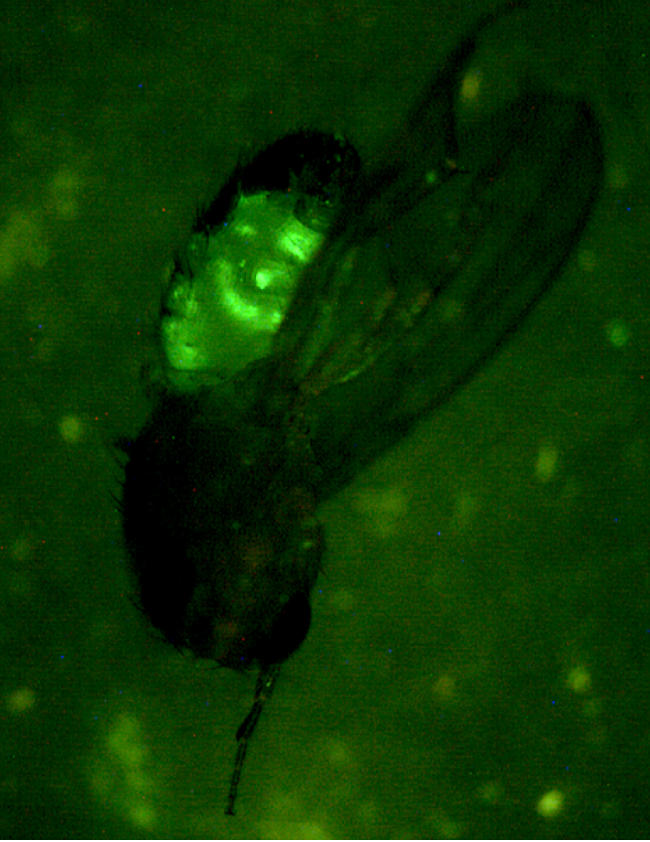
Adult fruit fly infected with flourescent Salmonella

Schneider and colleagues used a Salmonella strain (S. tyhphimurium) that produces two pathogenicity complexes, called type III secretion apparatuses, which shuttle virulence molecules through the host's cell membrane and into its cytoplasm. One complex, SPI1, facilitates cell entry while the other, SPI2, retools the intracellular environment to suit bacterial growth. The authors created a series of less virulent Salmonella strains and examined their effects on wild-type (nonmutant) flies. In addition, they looked at infections of wild-type Salmonella in flies carrying mutations in two critical immune response pathways (called Toll and imd, for immune deficiency).

Imd mutants infected with nonmutant Salmonella died much faster than the Toll mutants and had far more bacteria in their blood. When Schneider and colleagues correlated Salmonella population numbers with fly fate, they discovered something surprising. In other bacteria models, flies die when bacterial pathogen numbers reach a critical mass. Here, Salmonella populations hit a ceiling and the flies died with comparatively few bacteria. In flies infected with Salmonella strains lacking either one or the other virulence complex, the flies survived despite increased bacterial growth.

The authors explain this surprising finding with a model in which the fly's immune response produces substances that ultimately engineer its own destruction. Their model is supported by experiments showing that flies carrying a mutation in the *eiger* gene—a homolog of the human TNF gene—live longer with Salmonella infections. This is the same phenomenon seen in TNF-induced septic shock, when patients die as much from their immune system's response to infection as from the bacteria itself. Since many of the proteins involved in the Drosophila imd pathway have counterparts in the mammalian antibacterial immune response, the model described here can help identify the genetic agents of metabolic collapse associated with bacterial infections in humans.

